# Optimization of the Conditions of Solid Lipid Nanoparticles (SLN) Synthesis

**DOI:** 10.3390/molecules27072202

**Published:** 2022-03-28

**Authors:** Ewelina Musielak, Agnieszka Feliczak-Guzik, Izabela Nowak

**Affiliations:** Faculty of Chemistry, Adam Mickiewicz University, 8: Uniwersytetu Poznańskiego, 61-614 Poznan, Poland; ewelina.musielak@amu.edu.pl (E.M.); agaguzik@amu.edu.pl (A.F.-G.)

**Keywords:** solid lipid nanoparticles, optimization, formulation parameters, high-pressure hot homogenization, curcumin

## Abstract

Solid lipid nanoparticles (SLNs) have been synthesized as potential drug delivery systems. They are classified as solid lipid nanocarriers that can successfully carry both hydrophilic and hydrophobic drugs. SLNs are based on a biocompatible lipid matrix that is enzymatically degraded into natural components found in the human body. Solid lipid nanoparticles are suitable for the incorporation of hydrophobic active ingredients such as curcumin. The study included the optimization of lipid nanoparticle composition, incorporation of the active compound (curcumin), a stability evaluation of the obtained nanocarriers and characterization of their lipid matrix. Through process optimization, a dispersion of solid lipid nanoparticles (solid lipid:surfactant—2:1.25 weight ratio) predisposed to the incorporation of curcumin was developed. The encapsulation efficiency of the active ingredient was determined to be 99.80%. In stability studies, it was found that the most suitable conditions for conducting high-pressure homogenization are 300 bar pressure, three cycles and a closed-loop system. This yields the required values of the physicochemical parameters (a particle size within a 200–450 nm range; a polydispersity index of <30%; and a zeta potential of about |±30 mV|). In this work, closed-loop high-pressure homogenization was used for the first time and compared to the currently preferred open-loop method.

## 1. Introduction

The continuous progress in nanotechnology and its growing role in the world of science have led to the development of a new class of materials, which are lipid nanoparticles (LNPs). They make an interesting research object due to their potential applications in biotechnology, pharmaceuticals, optics and medicine. Lipid nanoparticles were first synthesized in the early 1990s, and their synthesis was groundbreaking in the search for a new generation of nanocarriers. They have been proven to enhance drug bioavailability, enable the controlled release of active ingredients, improve intracellular permeability, and enable the control of drug delivery to target sites through their surface modification capabilities [1]. They are defined as colloidal particles consisting of a solid lipophilic matrix into which active ingredients can be incorporated, and their size ranges from 40 nm to 1000 nm [2,3]. Typically, LNP carriers consist of a core of solid lipids along with a bioactive material that is an incorporated part of the lipid substrate. The whole structure is stabilized by a coating of surfactants (emulsifiers) and cosurfactants, if necessary [4]. There are two types of lipid nanoparticles: those of the first generation, which are solid lipid nanoparticles (SLNs), composed of solid fats only [5,6]; and those of the second generation, which include nanostructured lipid carriers (NLCs), containing in their structure not only solid but also liquid fats (oils). Both generations of lipid nanoparticles are composed of two immiscible phases: lipid and aqueous; therefore it is necessary to use surfactants for their synthesis [7,8]. Another difference in the structure of both particles is in the distribution of the active substance in the lipid matrix [9,10].

**Scheme 1:**
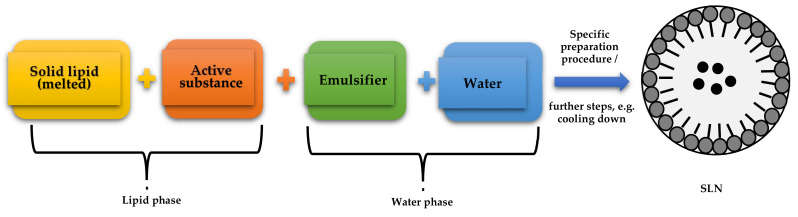
Block diagram of the structure of a solid lipid nanoparticle. Based on [17].

The best-known group of lipid nanoparticles is that of solid lipid nanoparticles, synthesized for the first time in 1991 [11]. They are colloidal lipid carriers that remain solid at room temperature and at human body temperature. These carriers are characterized by several functional advantages including high physical stability, composition based on lipids well absorbed by the human body, biocompatibility, and the lack of biotoxicity [12]. They usually have a spherical shape and particle diameter in the range of 50–1000 nm. Their small size and good application properties have prompted studies on the modification of the release of active ingredients and drugs [13]. Solid lipid nanoparticles are relatively easy to obtain and market due to the high availability of lipids [14]. However, their greatest advantages include non-toxicity, which is due to the biodegradable lipid structure [15]. A single solid lipid nanoparticle consists of an active ingredient along with a solid lipid (lipid phase) and an emulsifier with water (aqueous phase) (Scheme 1) [16].

The biodegradable lipid matrix formed in the process of SLN synthesis is enzymatically degraded into natural components found in the human body [18]. Thus, solid lipid nanoparticles can be successfully used as drug delivery systems (DDS) [16] as they can act as carriers for many active substances, including those of plant origin.

The first attempt at the isolation of plant active substances dates back to the early 19th century. It is estimated that about 70–95% of the population of highly developed countries still use medicines based on plant extracts in natural medicine [19]. Nowadays, medicinal herbs are defined as plants containing valuable active substances known to have therapeutic properties for the prevention and treatment of various ailments. A well-known and widely used plant-derived substance is curcumin (1,7-bis(4-hydroxy-3-methoxyphenyl)-1,6-heptadiene-3,5-dione). It is a hydrophobic polyphenol, isolated from the root of *Curcuma longa*. It was first extracted from turmeric in pure crystalline form in 1870 [20] and has since been widely known and used worldwide. In India, turmeric containing curcumin is used as a food additive; in Japan, it is served in tea; in Thailand it is used in cosmetic preparations; in China, it is currently used as a colorant; and in the United States, it is used in food industry as a preservative and colorant, among other applications. Curcumin is available in several forms, including capsules, tablets, ointments, soaps and cosmetics. It has been approved by the U.S. Food and Drug Administration (FDA) as “generally recognized as safe” [21]. Clinical studies have confirmed very good tolerability and safety profiles of curcumin at doses ranging from 4000 to 8000 mg/day [22], and even at doses up to 12,000 mg/day at 95% concentrations of the three curcuminoids: curcumin, bisdemethoxycurcumin and demethoxycurcumin [23]. This active ingredient and its derivatives have received much attention over the past two decades for their bifunctional properties such as antioxidant, anti-inflammatory, and most importantly, anticancer effects [24]. Curcumin is used in traditional medicines for a wide range of ailments including wound healing, urinary tract infections and liver problems. It has been known for its medicinal benefits for centuries, but it was not until 1937 that the first case of its use as a drug for the treatment of inflammatory disease was documented [25].

The aim of this work was to design the synthesis of solid lipid nanoparticles, to be used for the delivery of active ingredients. The effects of several factors were considered, including the type of lipid or surfactant and the method of preparation. Additionally, high-pressure homogenization (HPH) was chosen as the most effective and efficient method for the synthesis of lipid nanoparticles [26]. This method is more effective than the other well-known methods for SLN synthesis such as microemulsion, ultrasonic or solvent evaporation methods [27]. Additionally, we report here, for the first time, a new methodology based on the use of a closed loop in the HPH system. It is worth stressing that the optimization of SLN synthesis to achieve a homogeneous particle size distribution, high dispersion in the aqueous environment, long-term stability, and drug protection ability for designing improved drug formulations has not been presented to such an extent, and very few authors have attempted to explicitly discuss the sequential requirements for optimization [28].

The synthesis of SLNs, using HPH, begins with the preparation of a mixture. The solid lipid is melted and then an active ingredient (dissolved in the lipid phase) with specific pharmaceutical properties is added. Under continuous stirring on a magnetic stirrer, the lipid phase is dispersed in a hot aqueous surfactant solution. The temperatures of both the lipid and aqueous phases should be close and within 5–10 °C above the melting point of the solid lipid. The combined phases (pre-emulsion) are subjected to high-speed homogenization (UT) to facilitate further homogenization processes. This results in a dispersed macroemulsion, which is then subjected to high pressure. For hot high-pressure homogenization, pressures in the range of 500–1000 bar are most commonly used, and the entire process is carried out in at least three cycles. In the final step, due to cooling of the nanosuspension, its droplets crystallize and, thus, form solid lipid nanoparticles [29]. The SLN synthesis method used can be optimized at multiple stages of the process to obtain the best physicochemical properties of the resulting materials. To conclude, optimization strategies are procedures by which optimal process parameters are found. The effects of many factors or variables were taken into account, as were the amount of solid lipid, the amount of surfactant, etc. [30].

## 2. Result and Discussion

### 2.1. Selection of Lipids

The ratio of all the lipids tested in combination with curcumin was 1:100. All the prepared mixtures were observed at specific time intervals, and the results are shown in Table 1.

The table above (Table 1) shows the miscibility of curcumin in each of the lipids tested. It was observed that during the first 15 min above the melting point of each lipid, curcumin was mixed in only two lipid substances: Imwitor^®^ 900 K and Softisan^®^ 601. After 30 min, no change was observed, and homogeneous mixtures were obtained. This effect persisted over the next 24 h and then up to 72 h. As a result, Softisan^®^ 601 and Imwitor^®^ 900 K showed the highest compatibility with curcumin.

On the basis of the above results, two of the lipids tested passed the lipid screening. Therefore, they were used in the preparation of solid lipid nanoparticles. Nanoparticles based on both Softisan^®^ 601 and Imwitor^®^ 900 K were synthesized under the same conditions. 

A dynamic light scattering (DLS) method was used to determine the average size (Z-Ave) of the solid lipid nanoparticles obtained. With this method, it is possible to estimate the particle size in the range of a few nanometers to about three microns, and it is considered to be the most effective technique used to determine the size of nanoparticles [31]. Another size-related parameter is based on the so-called polydispersity index (PDI) determination, whose value should be in the range of 0–30% (0–0.3) for stable emulsions. The PDI parameter describes the degree of dispersity of lipid nanoparticle dispersions [32] and is denoted by Đ. It relates to the molecular weight and to the degree of polymerization and is calculated by dividing the average molecular weight (*M_w_)* by the molecular weight (*M_n_*), as expressed by the following formula Equation (1), Formula for calculating dispersity [32].):

Đ = *M_w_/M_n_*. (1)

**Figure 1:**
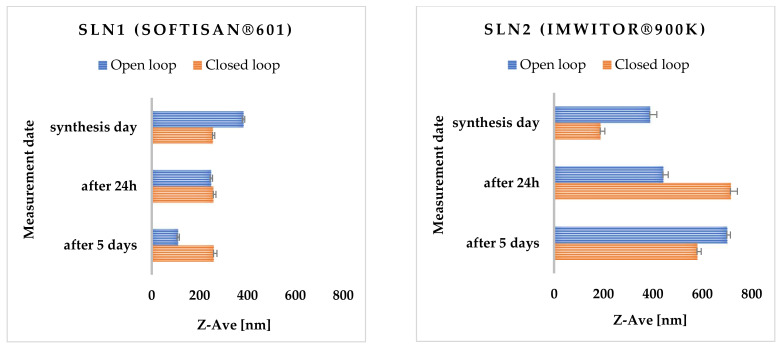
Comparison of the average particle size (Z-Ave) of the obtained SLNs synthesized using lipids Softisan^®^ 601 and Imwitor^®^ 900, and the surfactant Poloxamer 188, measured on the day of synthesis, and additionally 24 h and 5 days after the synthesis. The process of obtaining nanoparticles was carried out in an open-loop as well as a closed-loop system, under 300 bar pressure in three cycles.

The higher the degree of dispersity, the greater the mass scattering, so it is important to achieve a minimum PDI value [33]. The last important parameter determining the potential stability of the obtained lipid nanoparticles is the zeta potential (ZP). It is used to analyze the electrostatic properties of suspensions and determines the behavior of particles in a suspension. The zeta potential is denoted by the symbol ζ and is expressed in millivolts [mV]. It is often translated as the electrical potential that occurs at the so-called slip plane, which is the boundary of the electrical double layer of the particle in contact with the surrounding electrolyte [34]. The resulting ZP value indicates the degree of electrostatic repulsion between neighboring similarly charged particles in the SLN dispersion [35]. In order to avoid particle aggregation, and thus, ensure the absence of flocculation, the particles of the tested suspension must undergo repulsion [33,36]. In Table S1 from Supplementary Materials the values of Z-Ave, PDI and ZP are shown, measured immediately after synthesis, 24 h and 5 days after nanoparticle synthesis are presented. All SLN samples were stored at 25 °C. As shown in the analysis of the results, the most suitable data were obtained for SLNs containing Softisan^®^ 601 as lipid. The most favorable loop for this type of modification turned out to be the closed-loop system, a new method proposed by us. The average particle size just after the synthesis of unmodified SLN1 ranged from 255.50 ± 7.88 to 383.60 ± 2.60 nm, and after 5 days of storage in both cases, it was larger than immediately after the synthesis; this could indicate a gradually developing particle agglomeration (Figure 1).

**Figure 2:**
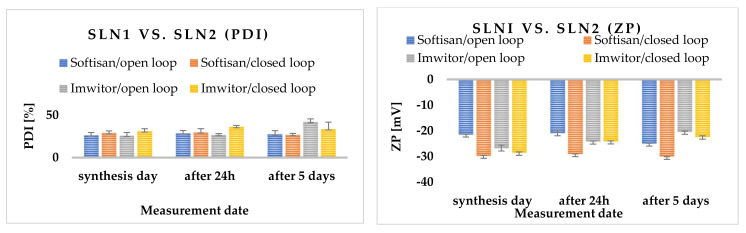
Comparison of polydispersity index (PDI) and zeta potential (ZP) of SLNs synthesized using lipids: Softisan^®^ 601 and Imwitor^®^ 900 and the surfactant Poloxamer 188 measured on the day of synthesis, and additionally 24 h and 5 days after the synthesis. The process of nanoparticle preparation was carried out in both open- and closed-loop systems under 300 bar pressure in three cycles.

The polydispersity index for Softisan^®^ 601 did not exceed (PDI) < 30%. Measurements for Imwitor^®^ 900 K showed that the threshold of acceptable PDI was exceeded on the last day of measurements for both the open- and closed-loop system. Zeta potential values ranged from approximately |±20.40| ± 0.30 to |±20.10| ± 0.06 for all SLNs analyzed both immediately after synthesis and after 24 h and 5 days of sample storage. Over time, the appearance of an irreversible flocculation process was observed, which was caused, among other things, by the change in particle size [37]. Flocculation could have been enhanced by the presence of a surfactant (Poloxamer 188), which has concentration-dependent destabilizing and stabilizing properties [38]. To prevent the flocculation process in SLN dispersions, a cosurfactant should be added [39]. It is also worth noting the effect of zeta potential on the stability of the resulting nanoparticles. Dispersions of solid lipid nanoparticles are considered stable if electrostatic repulsion prevails over van der Waals forces, while the absence of electrostatic repulsion (due to low ZP values) favors particle agglomeration [40] (Figure 2).

The stability of solid lipid nanoparticles is related to both particle migration and the resulting instability processes (e.g., creaminess, sedimentation, flocculation) and changes in particle size distribution that occur due to particle interactions [41]. Estimating the long-term stability of SLNs is of utmost importance when using these types of carriers for therapeutic purposes. Stable SLN dispersions are mainly characterized by maintaining a constant particle size, polydispersity index and zeta potential. A high PDI value usually favors crystal growth and the phenomenon of Ostwald ripening [26,42], the so-called isothermal crystallization. Ostwald ripening is a thermodynamic process that occurs in saturated systems in which smaller particles deposit on the surface of larger particles and recrystallize, resulting in the re-growth (“maturation”) of large droplets at the expense of smaller ones [43].

### 2.2. Surfactant Selection

Solid lipid nanoparticles are colloidal particle systems stabilized by surfactants. To select a suitable surfactant, nanoparticles were prepared with six different surfactants using Softisan^®^ 601 as the lipid, then their particle sizes were evaluated on the basis of PDI and zeta potential (ZP). The results obtained are shown in Table S2 from Supplementary Materials. The surfactant selection studies were conducted only in closed-loop system using hot high-pressure homogenization.

As shown in the results, the use of Poloxamer 188 (Pluronic F 68) resulted in a PDI of 26.70 ± 1.70% and a mean particle size of 259.50 ± 12.80 nm. Poloxamers are non-ionic triblock copolymers which consist of a central hydrophobic polyoxypropylene (poly (propylene oxide)) chain surrounded by two hydrophilic polyoxyethylene (poly (ethylene oxide)) chains. These surfactants sterically stabilize the nanoparticles and reduce the adsorption of plasma proteins by endowing the nanoparticle surface with hydrophilic properties and, thus, may prevent the removal of the active substance incorporated into SLNs from the bloodstream [44]. It has been reported that large and non-ionic surfactants are less toxic than single-chain and ionic surfactants [45]. Therefore, Poloxamer 188 was selected for further synthesis of SLNs. 

**Figure 3:**
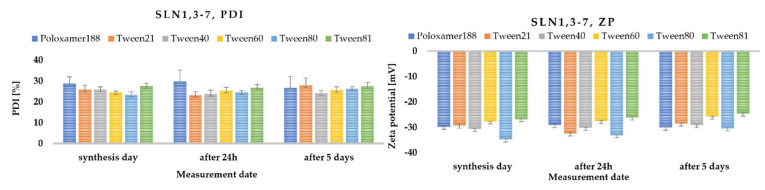
Comparison of polydispersity index (PDI) and zeta potential (ZP) of SLN synthesized with Softisan^®^ 601 lipid, and with all available surfactants (Poloxamer 188, Tween 21, Tween 40, Tween 60, Tween 80 and Tween 81), measured on the day of synthesis, and additionally 24 h and 5 days after synthesis. The process of obtaining nanoparticles was carried out in a closed-loop system under 300 bar pressure in three cycles.

The PDI ratio for all surfactants was within the upper limit of 30%; moreover, even after several days of synthesis, it did not exceed this value. The zeta potential decreased slightly with time, which may indicate that the non-ionic surfactants used formed a stable layer on the surface of the solid lipid nanoparticles (Figure 3). Figure 3 also shows the ZP values (in Milli-Q^®^ Plus water at pH = 5.50 and a conductivity of 50 μS/cm, and in aqueous surfactant solution), which were obtained immediately after synthesis, and 24 h and 5 days after the nanoparticle synthesis.

### 2.3. Method Selection

Various critical process variables that can significantly affect the quality of the synthesized solid lipid nanoparticles were also selected.

#### 2.3.1. Pressure Variation

**Figure 4:**
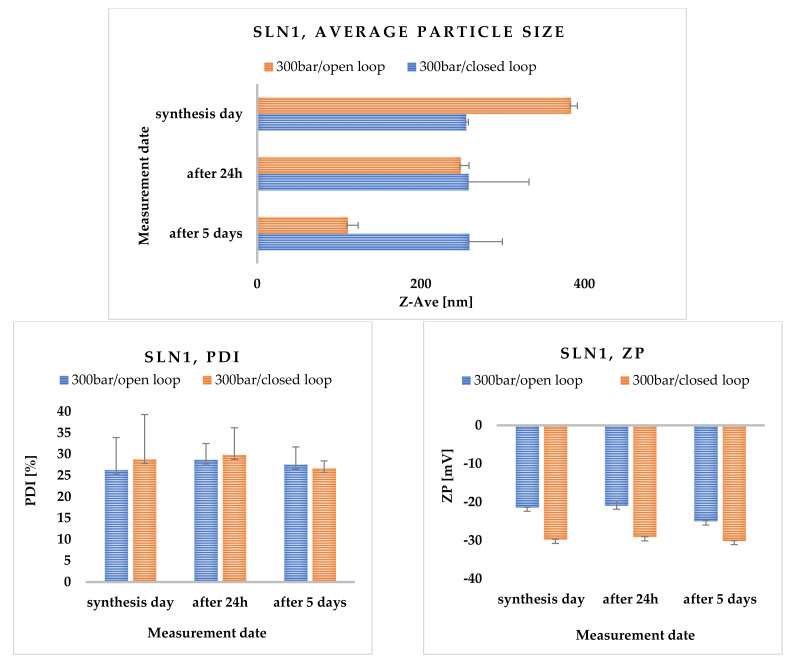
Comparison of average particle size, polydispersity index (PDI) and zeta potential (ZP) of nanoparticles synthesized with Softisan^®^ 601 lipid and Poloxamer 188 at 300 bar, measured on the day of synthesis, and additionally 24 h and 5 days after the synthesis. The process of obtaining nanoparticles was carried out in an open- and closed-loop systems in three cycles.

Preliminary optimization was carried out experimentally at three different pressures: 300, 400 and 500 bar. All the results obtained are summarized in Table S3 from Supplementary Materials. According to the results, as the homogenization pressure increases, the particle size increases and the polydispersity index (PDI) increases, which may be due to the formation of foam during high-pressure homogenization and more intensive aggregation in the formulation. The best results were obtained at 300 bar pressure. Hence, further studies were conducted at this pressure (Figure 4).

#### 2.3.2. Sonification

Particle size, zeta potential and PDI were determined to investigate the effect of sonification prior to the high-pressure homogenization process. The obtained emulsions were subjected to ultrasonication in three time duration variants: at 1 min, 5 min and 10 min (Table S4 from Supplementary Materials). The initial process was followed by hot high-pressure homogenization at 300 bar.

Analysis of the compiled data shows that an additional treatment with ultrasound (sonification) is not beneficial because the average particle size increases from 68.80 ± 7.30 nm up to 342.50 ± 22.10 nm with time, which may indicate rapid agglomeration of the particles. Another negative factor is the lack of homogeneity of the synthesized nanoparticles. On the basis of the zeta potential measurements, it can be seen that the stability of the obtained nanoparticles is low. The zeta potential on the day of synthesis was very low and decreased with time.

#### 2.3.3. The Effect of Sonification and High-Speed Homogenization (UT)

In addition to using sonification or the Ultra-Turrax high-speed homogenizer separately (Ystral GmbH, Ballrechten-Dottingen, Germany), their combined use was also tested. Both methods were used before high-pressure homogenization to prepare the pre-emulsion. After the lipid and aqueous phases were combined, the solution was treated with Ultra-Turrax (Ystral GmbH, Ballrechten-Dottingen, Germany), then the mixture was placed in an ultrasonic bath for 10 min (Table S5 from Supplementary Materials). 

The combination of methods did not result in significant changes. Similarly, as for the sonification, the particles were characterized by widely varying sizes and the lack of homogeneity. The PDI coefficient increases with time, which indicates the beginning of the particle agglomeration process. Zeta potential is very low and decreases even more with time, which indicates the lack of stability of the synthesized particles.

#### 2.3.4. Influence of the Number of High-Pressure Homogenization Cycles

Preliminary optimization of the number of cycles in the high-pressure homogenizer was carried out experimentally for four variants: three, five, six and seven cycles (Table S6 from Supplementary Materials). Measurements were carried out only on the day of synthesis and on the 5th day after the synthesis. As shown in the particle size and PDI results obtained, with increasing time of homogenization, the particle size increased, which may be attributed to the agglomeration process. It was also observed that as the number of cycles during the process changed and the polydispersity index increased, which may be due to foam formation and greater aggregation in the formulation. The best results were obtained for three cycles.

#### 2.3.5. Effects of Open- and Closed-Loop System

The optimization of lipid nanoparticle synthesis parameters was performed for both a closed- and open-loop system. As can be seen from the data obtained, the use of a closed loop proved to be much more advantageous in terms of long-term stability of the obtained solid lipid nanoparticles. Such results are influenced by the constant conditions of the hot high-pressure homogenization process, i.e., pressure, temperature, and controlled synthesis time. 

It was also observed that the obtained nanoparticles exhibit much better polydispersity (PDI) and zeta potential (ZP) parameters. To our knowledge, such closed-loop syntheses have not been carried out so far. 

#### 2.3.6. Physicochemical Characterization of Solid Lipid Nanoparticles 

In order to characterize the solid lipid nanoparticles, the average particle size, polydispersity index (PDI) and zeta potential (ZP) were measured (Table 2).

**Figure 5:**
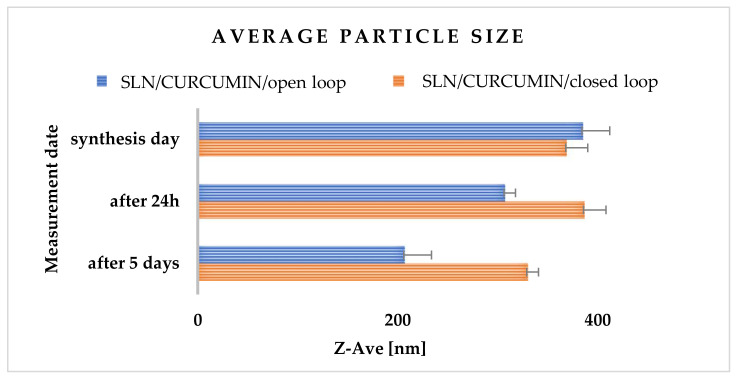

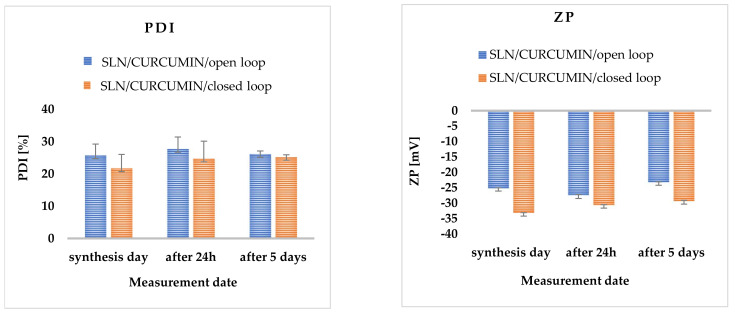
Comparison of the average particle size, polydispersity index (PDI) and zeta potential (ZP) of SLN-type lipid nanoparticles synthesized at 300 bar and incorporated with curcumin, measured on the day of synthesis, 24 h after synthesis and 5 days after synthesis, for a closed-loop system. The process was carried out in an open-loop and a closed-loop system in three cycles.

According to the results collected in Table S2 from Supplementary Materials, the average particle size just after the synthesis of SLNs incorporated with curcumin (SLN19) for 300 bar ranged from 206.90 ± 26.70 nm to 406.10 ± 52.60 nm. This shows a high degree of homogeneity of the curcumin-incorporated nanoparticles. Thus, it was inferred that the encapsulated active ingredients, such as curcumin, may reduce the size of the obtained lipid nanoparticles to some extent. After 24 h of storage of all emulsions, it was observed that the average particle size in all samples was smaller than immediately after the synthesis. The most favorable results were obtained for nanoparticles with curcumin synthesized at 300 bar (Figure 5).

##### X-ray Diffraction (XRD)

X-ray diffraction (XRD) data provide information on the degradation that occurs in crystalline and/or amorphous regions of the sample [46]. It is known that the process of crystallization can affect the degradation rate of the lipid matrix and thus the kinetics of the drug/active substance release [47]. In general, a drug is released much slower in amorphous form than in crystalline nanoparticles [46,48]. Lipid nanoparticles are made up of the lipids that can exist in various polymorphic forms, related to the many possible arrangements of aliphatic chains [49,50]. The polymorphic structure of lipids not only affects the release of the drug/active substance during storage but also the encapsulation efficiency [50]. The X-ray diffraction (XRD) technique permits identification of the structure of the obtained nanoparticles on the basis of comparing the obtained diffractograms with those of the corresponding standard.

According to the literature, lipids may exist in the form of three polymorphic forms: α, β or β′ [50,51]. The α form is referred to as unstable (amorphous), while β is considered to be the most stable form. The β′ form is known as metastable, characterized by a little disorder but still maintaining a partly amorphous state [51]. All forms are characterized by different packing of subcellular lipid chains, different angles of the lipid chains with respect to the glyceride molecular layers, and different densities. The different packings lead to characteristic wide-angle X-ray reflections suitable for structure identification. For saturated monoacylglycerols, α and β forms are observed in the unsaturated state, as well as in colloidal dispersions, whereas the β′ form is usually found only under special conditions. The β′ modification is often observed in more complex triacylglycerols, their mixtures, and in compositions containing larger fractions of partial glycerides (e.g., in many hard lipids or glycerol behenate). Using the X-ray diffraction method, the presence of the β′ form in colloidal particles has been confirmed [51]. All SLN samples tested were synthesized using the same lipid (Softisan^®^ 601). When analyzing the results, special attention was paid to the sample containing curcumin, as it could have altered the polymorphic form of the lipid used, changing the properties of the whole nanoparticle. 

**Figure 6:**
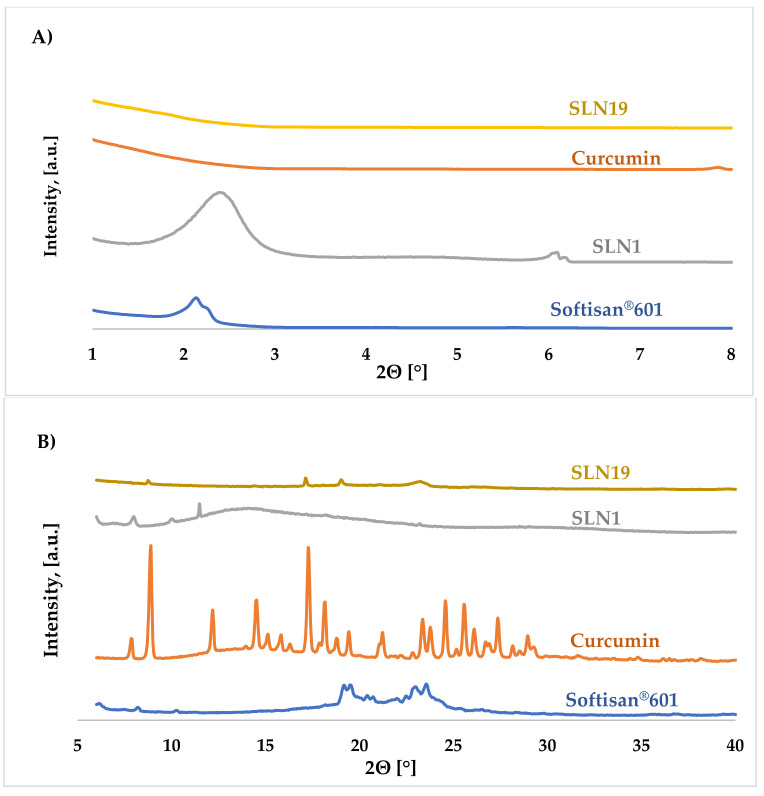
Comparison of XRD diffractograms: (**A**) low-angle diffractograms for Softisan^®^ 601, SLN1, curcumin and SLN19, and (**B**) wide-angle diffractograms for Softisan^®^ 601, SLN1, curcumin, and SLN19.

Figure 6A shows the low-angle diffractograms of Softisan^®^ 601, unmodified (“empty”) SLN1, curcumin, and SLN19 (incorporated with curcumin), while Figure 6B shows the wide-angle diffractograms of the same materials.

The results obtained in the low-angle range implied that the incorporation of curcumin into solid lipid nanoparticles did not disturb the structure of the whole particle. For the reference sample (SLN1), a characteristic reflection can be observed within the 2θ = 2.00–2.50° angle originating from the lipid. For the SLNs incorporated with curcumin, a small signal in this range was observed. The sample of solid lipid nanoparticles could adopt the metastable β’ form. From the obtained high-angle diffractograms, one small, low-intensity reflection was observed for curcumin-embedded SLNs. The highest diffraction intensity was obtained for a 2θ angle of 23.50° (SLN19). For the SLN1 sample, one broad reflection visible within the 2θ angle of 13.38–15.12° was noted. It was concluded that both the reference sample (SLN1) and SLN19 have a slightly ordered structure and occur in the β’ form.

##### Differential Scanning Calorimetry (DSC) 

Differential scanning calorimetry (DSC), a thermoanalytical technique, is based on maintaining the same temperature for a test sample and a reference sample, and measuring the difference in the heat flux delivered to the two samples. With this technique, it is possible to measure the difference between the heat flows that occur while the sample absorbs or releases heat due to thermal effects, such as melting, crystallization, chemical reactions, polymorphic transformations, or the evaporation process [52]. Heat exchange during temperature-controlled measurements provides information regarding the structural properties of the sample. By using differential scanning calorimetry, it is possible to measure the enthalpy (ΔH) and the change in heat capacity (ΔC_p_) of thermal effects [53].

**Figure 7:**
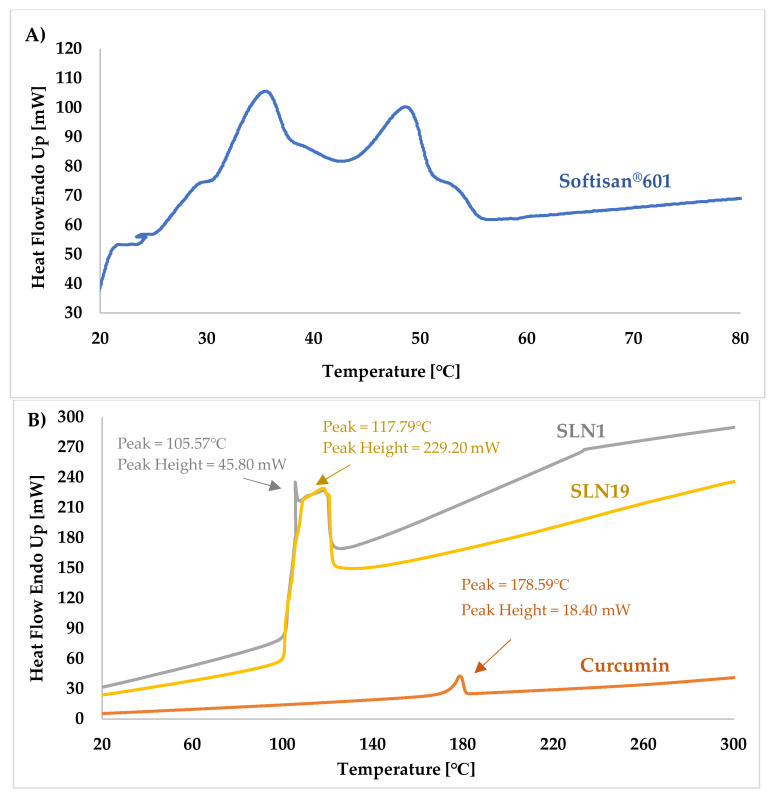

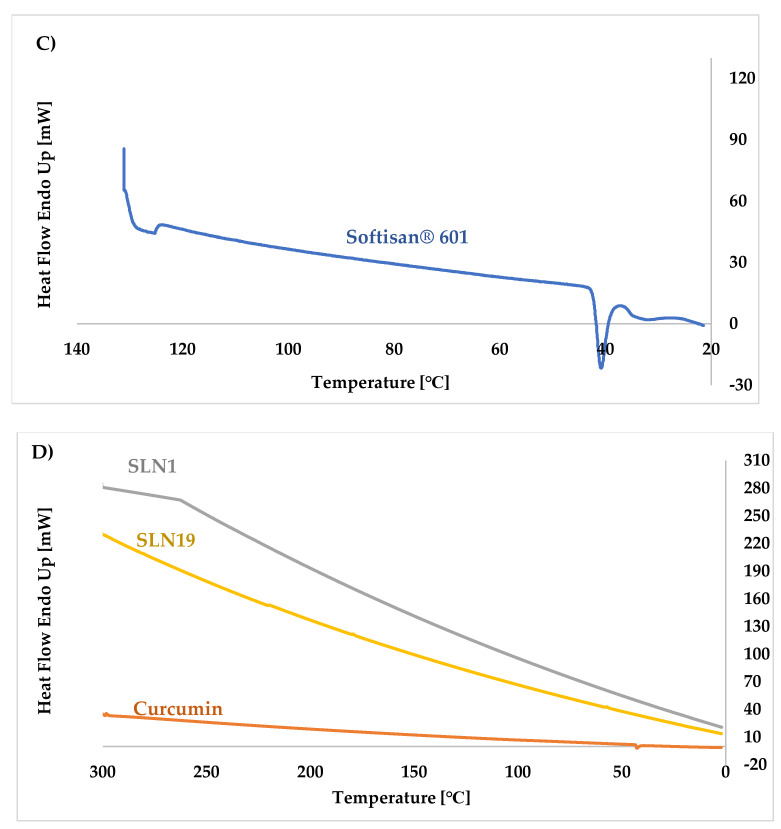
Comparison of DSC thermograms recorded (**A**) upon heating of Softisan^®^ 601 from 25 to 80 °C, and (**B**) upon heating of the curcumin, SLN 1, and SLN 19 from 25 to 300 °C; and comparison of DSC thermograms recorded (**C**) upon cooling of Softisan^®^ 601 from 140 to −20 °C and (**D**) upon cooling of curcumin, SLN 1, and SLN 19 from 300 °C to −20 °C.

Figure 7A,B show a comparison of the thermograms of the SLN samples—both unmodified (“empty”) (SLN1, Figure 7B) and those modified with incorporated curcumin (SLN19, Figure 7B)—and the reference samples (i.e., Softisan^®^ 601 (Figure 7A) and curcumin (Figure 7B)) during DSC heating; Figure 7C,D show the results recorded on cooling of the same samples. 

The DSC thermograms recorded upon heating of sample SLN19 (from 25 to 300 °C) show the first intense exothermic peak, associated with crystallization (solidification) of the lipid matrix [52], at about 117.79 °C. For the unmodified nanoparticles (SLN1), the intense exothermic peak was observed at about 105.59 °C, and for curcumin at 178.59 °C. The analysis of the obtained results shows that the samples with curcumin in the lipid matrix of the nanoparticles were characterized by a higher solidification temperature than those devoid of the encapsulated active ingredient [52].

At the initial stage of the crystallization process, i.e., at 105.59 °C, the most intense exothermic peak was observed for the reference sample (SLN1), for which its intensity was 45.80 mW, for SLN19. On the other hand, the highest peak was observed at 229.20 mW.

In the DSC thermograms recorded upon cooling of SLN19, the reference sample (SLN1) and curcumin, no visible endothermic peaks were observed; this is attributed to the initial phase of matrix breakdown and oxidation of the encapsulated substance [52,53]. The absence of visible peaks in these thermograms indicates that the lipid matrix, together with the active ingredient, did not disintegrate. The analysis of DSC recorded upon heating (from 25 to 300 °C) and cooling (from 300 to −25 °C) permitted comparison of the types and temperatures of the thermal transformations taking place in the samples. Thus, it was deduced that in all the analyzed samples, melting and crystallization processes occurred, and that the melting process started first (ΔH > 0; endothermic reaction) and was followed by the crystallization process (ΔH < 0; exothermic reaction). Moreover, the DSC analysis of lipid nanoparticles was particularly concerned with the study of thermal phenomena taking place on the lipid (included in the SLN). 

Differential scanning calorimetry provided information on the characteristic temperature parameters of individual samples, which may improve the quality and physicochemical properties of cosmetic/pharmaceutical products during further technological processes using lipid nanoparticles incorporated with curcumin.

##### Scanning Electron Microscopy (SEM) 

By using scanning electron microscopy, it was possible to obtain images of the studied materials at magnifications of 20, 10 and 2 μm (Table 3). The surface structure and porosity of the materials were observed through the SEM images. 

Table 3 shows the scanning electron microscopy SEM images of pure curcumin, SLN1 and SLN19. Image magnification allowed the observation of particles on a scale from 2 to 20 μm. The images show agglomerations of lipid nanoparticles of irregular shapes and surfaces, as well as powder of curcumin for the sake of comparison. Exemplary lipid nanoparticles, unmodified (“empty”) and with incorporated curcumin, show morphological irregularities and surface roughness, which is consistent with the literature data [54,55,56]. The visible imperfections may be a consequence of prolonged exposure of the samples to a high vacuum and an intense electron beam. No significant differences in morphology were observed between the reference sample (SLN1) and the sample with the incorporated curcumin (SLN19). The images presented here show that the high-pressure homogenization method produces a polydisperse SLN formulation with a wide size distribution. As can be seen, some particles formed large “clusters” (agglomerates). This is a phenomenon characteristic of SLNs, which show a high tendency to form a gel and to aggregate due to the “sticky” nature of the lipid, especially after long-term storage.

##### Confocal Microscopy

By using confocal microscopy, it was possible to observe the autofluorescence of the active ingredient, that is curcumin. By monitoring the sample with confocal microscopy, the incorporation of the active ingredient into solid lipid nanoparticles was confirmed. 

Confocal microscopy images for pure curcumin, SLN1 and SLN19 are shown below.

Curcumin possesses fluorescent properties. Thus, the autofluorescence changes observed above prompt us to understand the localization of curcumin in lipid nanoparticles. Table 4 shows the images obtained using confocal microscopy for pure curcumin, SLN1 and SLN19.

As shown in the images, the highest intensity of autofluorescence was observed for pure curcumin. The above presented changes in curcumin autofluorescence permit evaluation of the distribution of its particles (localization) in lipid nanoparticles. For the samples of lipid nanoparticles with incorporated curcumin, the active substance was found to be homogeneously distributed. Thus, according to the results, curcumin as a lipophilic particle should easily bond to the solid lipid nanoparticles.

#### 2.3.7. Assessment of Encapsulation Efficiency and Loading Capacity of Curcumin

Encapsulation efficiency (EE) and loading capacity (LC) are key factors that determine the appropriate choice of qualitative and quantitative composition, and the method of preparation of lipid nanoparticle dispersions [57,58]. For each obtained lipid nanoparticle formulation, it is important to obtain the highest possible EE and LC values; this is because their values determine the mode of release of the active substance from the lipid matrix, and the target effectiveness of the obtained lipid nanoparticle dispersions containing the tested active compounds [6,59,60]. 

The results obtained from the evaluation of the encapsulation efficiency and loading capacity of curcumin in the lipid matrix are presented in Table 5.

The encapsulation efficiency of curcumin (84.52%) and the loading capacity (12.89%) were evaluated as expected, i.e., very good. This fact confirms the nature of the incorporated curcumin, which is a hydrophobic compound and, thus, has a high affinity for the matrix of solid lipid nanoparticles.

#### 2.3.8. Study of the Release Kinetics of Curcumin

A controlled release of active substances from pharmaceutical compounds is important for the evaluation of their efficacy. General guidelines for release studies of active substances can be found in *Pharmacopoeia*. It is important to determine parameters such as the type, volume and temperature of the receiving medium; agitation rate; sampling time; and concentration analysis of the active compound (by UV–Vis spectroscopy or HPLC chromatography) [61]. The subject of this study was the release of curcumin from cosmetic formulations (hydrogel, *o/w* emulsion) into a diffusion chamber connected to a UV–Vis spectrophotometer. The application of UV–Vis spectroscopy in release studies makes it possible to determine the kinetics of release of a bioactive substance from the formulation to the receiving medium [62]. The obtained release profiles were determined to be classical, indicating a gradual release of the active substance from the cosmetic substrate over time. 

**Figure 8:**
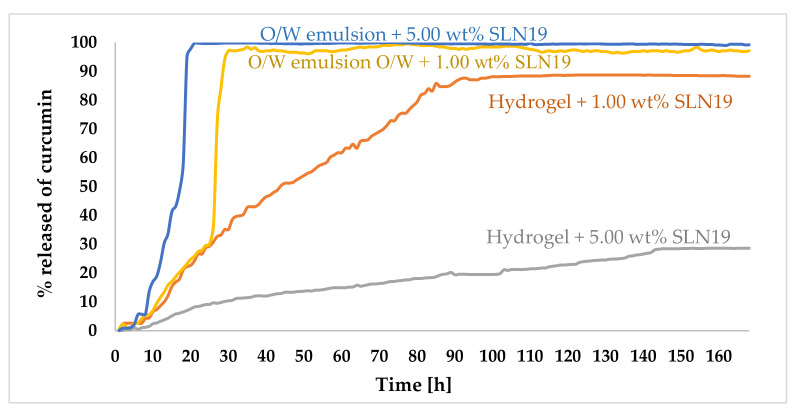
Release profile of curcumin from cosmetic formulations: hydrogel 1.00 wt.% SLN19; hydrogel 5.00 wt.% SLN19; emulsion *o/w* 1.00 wt.% SLN19; and emulsion *o/w* 5.00 wt.% SLN19 in phosphate buffer pH 5.8.

The release profiles of curcumin from hydrogel and *o/w* emulsion show that there are no substances in the formulation that could inhibit the diffusion of the active ingredient. The release process of curcumin was fast for the sample based on *o/w* emulsion with 1.00 wt.% and 5.00 wt.% SLN19 (Figure 8).

For the emulsion with 5.00 wt.% SLN19 in the first five hours of analysis, the release process was relatively slow. However, after the fifth hour, the release process increased rapidly, and as much as 99.80% of the active ingredient was released within 19 h from the beginning of the observation. A similar situation also occurred for the emulsion with 1.00 wt.% SLN19. However, a sudden increase in the percentage of released curcumin occurred at 22 h after the beginning of the analysis, and the maximum amount of active substance released was 97.20%. The release of curcumin from the hydrogel at both concentrations of SLN19 (1.00 wt.% and 5.00 wt.%) occurred gradually. Due to the slow release process of the active ingredient, a prolonged release was observed, which is beneficial for pharmaceutical and cosmetic formulations. The maximum amount of curcumin released from the hydrogel was 87.00% for 1.00 wt.% SLN19, and 28.50% for hydrogel with 5.00 wt.% SLN19.

The in vitro release of the active substance is largely related to the process of diffusion from the formulation to the receiving medium. The results obtained using UV–Vis spectrometry showed that the release rate of the active substance is highly dependent on the type of pharmaceutical/cosmetic formulation. Considering the *o/w* type formulation, the addition of lipid nanoparticles increased the lipophilic nature of the substrate, which shows a slightly higher affinity for the incorporated nanoparticles. In the case of the hydrogel, the incorporation of the lipid nanoparticles changed the nature of the hydrogel to the physicochemical form of a lipogel (still remaining a hydrophilic substrate). The profiles were additionally fitted to distinct mathematical models to find out the mechanism of the drug release [63]. The release profile from *o/w* emulsions best fitted the first-order kinetics, whereas hydrogels followed the zero-order kinetics.

## 3. Materials and Methods

### 3.1. Materials

The solid lipids Compritol^®^ 888 ATO and Precirol^®^ ATO 5 were purchased from Gattefossé (Lyon, France), while Imwitor^®^ 900 K and Softisan^®^ 601 were purchased from Cremer Oleo GmbH & Co (Hamburg, Germany). KG. All surfactants used in this study, namely Poloxamer 188, Tween 21, Tween 40, Tween 60, Tween 80, and Tween 81 were purchased from Sigma-Aldrich Sp. z.o.o (Poznań, Poland). Curcumin (purity > 65%) was purchased from Sigma-Aldrich Sp. z.o.o Poznań, Poland. All other chemicals and solvents were of analytical purity and were used without further purification.

### 3.2. Methodology

#### 3.2.1. Optimization of SLN-Type Lipid Nanoparticle Synthesis

Solid lipid nanoparticles should be designed to meet specific requirements towards the intended product performance. For this purpose, a series of experiments was carried out to adjust all the necessary parameters for the synthesis of solid lipid nanoparticles, both unmodified (“empty”) and incorporated with an active substance. The main objective of this work was to obtain SLNs with the best possible parameters, i.e., suitable polydispersity index (PDI) < 30% [64] and nanoparticle size. 

For SLN production, a solid lipid, an emulsifier, water and an active ingredient were used. The lipids used can be triacylglycerols (tristearin), monoglycerides (Imwitor), fatty acids (stearic acid, palmitic acid), and steroids (cholesterol) and waxes (cetyl palmitate). Various surfactants (Pluronic F 68, F 127, etc.) were used to stabilize the lipid dispersion [31].

##### Lipid Screening

Prior to the studies conducted in this work, the so-called lipid screening was performed. The miscibility of the active substance in selected lipids was determined experimentally. All the lipids tested, along with their melting points, are listed in Table S7 from Supplementary Materials.

The selection of lipids was made with consideration of the properties of the active ingredient, curcumin. Portions of 1.00 g of each of the selected solid lipids, shown in Table 5, were weighed into 4 glass vials. Then, 0.01 g of curcumin was added to each vial as the active ingredient. The vials were placed on a magnetic stirrer and the contents were heated to a temperature above the melting point of each of the selected lipids. The melted lipid, along with the active ingredient (curcumin), was stirred until a homogeneous mixture was obtained. After this time, the obtained mixtures were cooled to 25 °C and observations were recorded at specific time intervals, viz: 15 min, 30 min, 1 h, 24 h, 72 h. The most suitable lipid was chosen on the basis of the coloration of the lipid/curcumin mixture at specified time intervals. It was assumed that curcumin can be incorporated into lipid nanoparticles when the solidified mixture is homogeneous and clear. The solid lipids that showed no precipitation of curcumin were selected for further studies.

##### Surfactant Selection

The choice of surfactant depends mainly on the route of administration and the application of the final products, which are lipid nanoparticles. The amount of the surfactant used to synthesize the suspension of solid lipid nanoparticles and their concentration directly affect the size and degree of polydispersity of the particles. From among the commercially available surfactants, 6 commonly used ones were selected for SLN synthesis (Table S8 from Supplementary Materials).

##### Selection of Synthesis Method

Solid lipid nanoparticles were prepared using hot high-pressure homogenization. It is the most effective method so far for the reproducible synthesis of both non-incorporated (“empty”) SLNs and those incorporated with active substances. The lipid phase containing the active substance was dispersed in a hot aqueous surfactant solution upon continuous stirring. The solution was then homogenized at a temperature above the melting point of the lipid, using a high-pressure homogenizer to form an *o/w* nanoemulsion, which was then cooled to room temperature.

On the basis of literature review and a series of trials, various critical process variables that can have a significant impact on the quality of the nanoparticles were identified. The process parameters—i.e., homogenization rate, homogenization time, pressure, pre-homogenization (Ultra-Turrax high-speed homogenizer, Ystral GmbH D-7801, Ballrechten-Dottingen, Germany), ultrasound treatment time and type of a homogenizing system (open/closed)—were optimized taking into account their effect on the size of the nanoparticles obtained, their polydispersity index (PDI) and their zeta potential. The synthesis of the desired carriers was carried out by changing one parameter while keeping the other parameters constant. Each formulation was prepared twice so as to compare the open- and closed-loop performance during the hot high-pressure homogenization process.

##### Synthesis of Non-Incorporated SLNs 

After a lot of syntheses and experimental procedures, the method of hot high-pressure homogenization was found to be the most effective for the reproducible synthesis of both non-incorporated (“empty”) and curcumin-incorporated SLNs. The reference samples were unmodified SLNs, i.e., nanoparticles containing no active ingredient (Table 6). The absence of the active ingredient (1.00% by weight) in the lipid phase of the SLN dispersion was supplemented with the same amount of a given lipid. 

Finally, to obtain the lipid phase, Softisan^®^ 601 in the amount of 1.50 g (3.00% *w/w*), heated to 50 °C, was first placed into beaker, followed by the addition of an aqueous surfactant solution containing 0.62 g (1.25% *w/w*) of Poloxamer and 47.87 mL (95.75% *w/w*) of distilled water, heated to the same temperature as the lipid phase. The pre-emulsion thus obtained was pre-homogenized using an Ultra-Turrax (Ystral GmbH D-7801, Ballrechten-Dottingen, Germany)^®^ T25 at 10.000 rpm. The hot high-pressure homogenization method was used for SLN synthesis. The lipid macroemulsion was homogenized 3 times at 300 bar.

##### SLNs Incorporated with Curcumin

After optimizing the method for the synthesis of SLNs incorporated with the active ingredient (curcumin), the final formulation and method for their preparation were selected (Table 7). Thus, the SLN formulation contained: 1.00%wt. of the active ingredient (curcumin), as well as 2.00 wt. of solid lipid, 1.25 wt.% of the surfactant, and 95.75 wt.% of water.

The lipid phase, consisting of a solid lipid and curcumin, and the aqueous phase containing a surfactant and water, were placed on a magnetic stirrer equipped with a thermocouple, to melt the lipid and combine the surfactant with the water. Both the lipid with curcumin and the surfactant with water were heated to an appropriate temperature (water and lipid phases must be at the same temperature). The synthesis of SLNs incorporated with curcumin was carried out using 3-fold high-pressure homogenization (HPH) at 300 bar. To increase the efficiency of the solid lipid nanoparticle synthesis process, the lipid pre-emulsion was prepared prior to HPH using an Ultra-Turrax^®^ T25 Digital high-speed homogenizer (Ystral GmbH D-7801, Ballrechten-Dottingen, Germany).

#### 3.2.2. Physicochemical Characterization of Basic Parameters Describing Lipid Nanoparticles

The adequate and proper characterization of SLNs is essential for quality control of the obtained nanoparticles. Important parameters to be evaluated are particle size, size distribution kinetics (zeta potential), degree of crystallinity, and lipid modification (polymorphism). The average particle size (Z-average, Average particle size Z, Z-Ave), polydispersity index (PDI) and zeta potential (charge on the surface of the dispersion) were determined using a zeta potential analyzer. 

##### Average Particle Size and Polydispersity Index

In order to determine the average particle size Z-average (Z-average, Average particle size Z, Z-Ave) and polydispersity index (PDI), the dynamic light scattering (DLS) method was used. Each sample was analyzed using a Litesizer 500 particle size analyzer, (Anton Paar GmbH, Warsaw, Poland) at 25 °C. The apparatus was able to measure the particle size range from 0.3 nm to 10 μm. The laser beam (λ = 658 nm, 40 mW) and a scattered light detector set at three angles of 15°, 90°, and 175° were used to precisely detect low-intensity scattered light signals that may originate from smaller particles. Prior to analysis, each SLN dispersion was diluted 100-fold in highly demineralized water (Milli-Q^®^ Plus, Millipore, Germany), then the prepared sample was placed in a measuring cell made of polystyrene. The cell was filled to a height between 10 and 15 mm. During one cycle, three measurements were performed for each of the Z-Ave values. After the measurements were completed, the arithmetic mean and standard deviation of the results were calculated.

##### Zeta Potential (ZP)

The zeta potential (ZP) was measured using a Zetasizer Nano Z (Malvern Instruments, Worcestershire, UK). Prior to the measurement, each SLN dispersion was diluted 100-fold in highly demineralized water (Milli-Q^®^ Plus). Subsequently, a minimum of 1 mL of the appropriately prepared sample was transferred by syringe into a U-shaped capillary cell (DTS1060), which was placed in the measuring chamber of the instrument after sealing. The ZP value was measured three times during one cycle and was then calculated by the system software using the Helmholtz–Smoluchowski equation. After the measurements were completed, the arithmetic mean and standard deviation of the results were calculated.

##### X-ray Diffraction (XRD)

The degree of crystallinity and the presence of polymorphic forms of the lipid matrix of the obtained lipid nanoparticles were determined, on the basis of analysis of the results of X-ray diffraction (XRD) patterns; these were obtained using a D8 Advance powder diffractometer with a Johansson monochromator (Bruker, Billerica, MA, USA; λ_Cu-Kα_ = 0.15406 nm). Sample preparation consisted of placing the tested dispersions of lipid nanoparticles in a Petri dish and drying them at room temperature. The obtained samples were analyzed in the following ranges: low angles (SAXD: 0.6–0.8°) with a scanning speed of 0.02°/3 s, and wide angles (WAXD: 6–60°) with a scanning speed of 0.05°/1 s.

##### Differential Scanning Calorimetry (DSC) 

The evaluation of the polymorphic forms of the lipid matrix of lipid nanoparticles was carried out on the basis of the results of thermal analysis, performed with a DSC 8500 differential scanning calorimeter (Perkin Elmer, Waltham, MA, USA). The instrument was calibrated prior to the measurements. Further, a 40 μL aluminum plate was filled with a lipid sample or a sample of the solid lipid nanoparticle dispersion under study. The sample was then gradually heated from 25 °C to 300 °C in a flow of nitrogen (20 mL/min) at a rate of 5 °C per minute. After reaching the set temperature, the sample was held at 300 °C for 1 min, then cooled to −20 °C at the above-mentioned parameters.

##### Scanning Electron Microscopy (SEM)

The morphology of the lipid nanoparticles was determined by scanning electron microscopy (SEM, Quanta FEG 250 (FEI, Hillsboro, Oregon, USA), under a low-pressure vacuum (70 Pa) and a 10 kV beam accelerating voltage. For this purpose, the samples were placed on a carbon-coated 400-mesh copper grid. Under the influence of the electron beam, the sample emitted different signals, which were received by the detector and processed into an image of the sample.

##### Confocal Microscopy

Before taking images using a confocal microscope (Zeiss Axiovert 200 M, Oberkochen, Germany), the obtained materials, i.e., unmodified (“empty”) SLNs and SLNs incorporated with curcumin, were applied to a coverslip. Autofluorescence of curcumin was excited using a 488 nm laser.

#### 3.2.3. Encapsulation Efficiency and Loading Capacity 

The encapsulation efficiency (EE) and loading capacity (LC) of curcumin in the resulting lipid nanoparticles were determined using a high-performance liquid chromatograph (Varian 920-LC, Agilent Technologies, Santa Clara, CA, USA) coupled to a UV–Vis detector. Chromatographic determination of curcumin in the lipid nanoparticle dispersion involved quantification of the unincorporated active ingredient present in the outer aqueous phase of the surfactant. The encapsulation efficiency (EE) and loading capacity (LC) of curcumin were calculated according to the Equations (2) and (3) [65], to calculate the encapsulation efficiency (EE) and loading capacity (LC) of curcumin in the obtained lipid nanoparticles.

(2) $$\mathrm{EE}\%=\frac{Total\;amount\;of\;drug\;added-unentrapment\;drug}{Total\;amount\;of\;drug\;added}\;\times \;100$$

(3) $$\mathrm{LC}\%=\frac{Total\;amount\;of\;drug\;added-Unentrapment\;drug}{Total\;drug+Total\;lipid-Unentrapment\;drug}\;\times \;100$$

The samples were prepared by measuring 1 mL of the tested lipid nanoparticle dispersion into Eppendorf Tubes^®^, placing them in the angle rotor of an MPW-350R laboratory centrifuge and spinning at 3000 rpm for 30 min. In the next step, an aliquot of 9 mL of distilled water was added to the aqueous solution of the external phase, separated by centrifugation, and the sample was shaken vigorously for about 5 min. The resulting solution was passed through a syringe filter with a pore size of 0.45 μm, and a 1.5 mL portion was transferred to a glass vial for HPLC analysis. Chromatographic analysis consisted of the determination of curcumin content in the test samples using the external standard method based on calibration curves generated by a validated HPLC method. Each test sample was subjected to a series of three measurements, and the arithmetic mean and standard deviation were determined from the measurement results. 

#### 3.2.4. Study of the Kinetics of Curcumin Release from Prepared Cosmetic Formulations

In this study, the release kinetics of curcumin from prepared cosmetic formulations (hydrogel, *o/w* emulsion) was investigated.

##### Preparation of Hydrogel Containing 1 wt.% SLN

Portions of 1.00 g of glycerol (Chempur, Karlsruhe, Germania) and 8.63 g of demineralized water were weighed into a glass beaker. The whole mixture was heated to 80 °C. Then, hydroxycellulose (Sigma-Aldrich) was gradually added in the amount of 0.25 g, all the while intensively mixing the obtained formulation with a glass dipstick. After obtaining gel consistency, the system was cooled to 40 °C, then Microcare^®^ SB (Thor GmbH) was added and the whole mixture was stirred for 5 min. In the next step, once the temperature was below 30 °C, the active ingredient, curcumin (Sigma Aldrich), was added to the hydrogel in an amount of 0.10 g (1 wt.%). The obtained formulation was stirred for 10 min to stabilize the system.

##### Preparation of Hydrogel Containing 5 wt.% SLN

Portions of 1.00 g of glycerol (Chempur, Karlsruhe, Germania) and 8.23 g of demineralized water were weighed into a glass beaker. The whole mixture was heated to 80 °C. Then, hydroxycellulose (Sigma-Aldrich, Poznań, Poland) was gradually added in the amount of 0.25 g, all the while under intensive stirring of the obtained formulation with a glass dipstick. After obtaining the gel consistency, the system was cooled to 40 °C, then Microcare^®^SB (Thor GmbH, Speyer, Germany) was added and the contents were stirred for 5 min. In the next step, once the temperature was below 30 °C, the active ingredient curcumin (Sigma-Aldrich, Poznań, Poland) was added to the hydrogel in an amount of 0.50 g (5 wt.%). The obtained formulation was stirred for 10 min to stabilize the system.

##### Preparation of an *o/w* Emulsion Containing 1 wt.% SLN 

The lipid phase components Creagel^®^ EZ (Creations Couleurs) and Alphaflow^®^ 20 (Creations Couleurs) were weighed into a glass beaker and mixed using a glass dipstick. An aqueous phase was then prepared containing 0.02 g Microcare^®^ SB (Thor GmbH), 7.23 g demineralized water, and 0.10 g active ingredient (curcumin, Sigma-Aldrich). The two phases were mixed by stirring vigorously until the desired consistency was obtained. The obtained formulation was then homogenized using YellowLine DI 25 homogenizer (IKA) at 13500 rpm.

##### Preparation of an *o/w* Emulsion Containing 5 wt.% SLN

The lipid phase components Creagel^®^ EZ (Creations Couleurs) and Alphaflow^®^ 20 (Creations Couleurs) were weighed into a glass beaker and mixed using a glass dipstick. An aqueous phase was then prepared containing 0.02 g Microcare^®^ SB (Thor GmbH), 6.83 g demineralized water and 0.50 g active ingredient (curcumin, Sigma-Aldrich). The two phases were mixed by stirring vigorously until the desired consistency was obtained. The obtained formulation was then homogenized using YellowLine DI 25 homogenizer (IKA) at 13500 rpm.

##### Release Kinetics of Active Substance from Above-Mentioned Cosmetic Formulations

A 708-DS diffusion apparatus (Agilent Technologies, CA, USA) coupled with a Varian Cary 50 Bio UV–Vis spectrophotometer was used to evaluate the release kinetics of curcumin from the obtained formulations. The process temperature was maintained at 37 °C. A special cuvette was filled with 3.00 g of hydrogel or *o/w* emulsion containing an appropriate weight percentage of solid lipid nanoparticles (SLN) incorporated with curcumin, then a membrane mimicking the skin barrier (membrane made of a natural polymer, which is a special type of cellulose called Cuprophan) was placed over the sample surface. The studies of curcumin release were carried out in a phosphate buffer of pH 5.80. The process of the evaluation of curcumin release kinetics was carried out for 168 h. The amount of released active substance was calculated according to the following Equation (4):

(4) $$\%\;\mathrm{released}=(\frac{A_{p}}{A_{w}})(\frac{m_{w\;}\left[mg\right]\times C_{w}}{V_{w}\left[ml\right]})(\frac{1}{D_{w}})(\frac{V_{p}\;\left[ml\right]}{m_{p}\left[mg\right]})\times 100$$

where: *A_p_*—sample absorbance; *A_w_*—standard absorbance (absorbance of a standard solution prepared from pure curcumin and ethanol at a ratio of 1:250 *v/v*); *m_w_*—standard weight (weight of standard weighed to prepare a standard solution for UV–Vis measurements); *m_p_*—label content (content of the active substance inside the carrier); *C_w_*—standard purity; *D_w_*—dilution factor; *V_w_*—standard volume; *V_p_*—medium volume (phosphate buffer). 

The quantitative evaluation of released curcumin by UV–Vis spectrophotometry, like most instrumental analyses, is a comparative technique. Therefore, the analyzed sample was compared with a suitable standard solution prepared from pure curcumin (>90%) and 96% ethanol at a ratio of 1:250 *v/v*. The reliability of the quantitative assessment was confirmed by selecting an appropriate wavelength characteristic only of the substances analyzed and not of the receiving medium used (e.g., phosphate buffer). In the case of curcumin, the UV spectrum was recorded at 425 nm.

## 4. Summary and Conclusions

Among the well-known substances of plant origin, curcumin deserves special attention. Due to its high biological activity, curcumin has long been used in the pharmaceutical industry, cosmetics, and medicine. However, because of its low bioavailability, as well as rapid biodegradation, encapsulating it into solid lipid nanoparticles has proven to be an ideal method to improve the stability and efficacy of SLNs. Based on the present study, the synthesis of unmodified solid lipid nanoparticles and those encapsulated with curcumin was successfully optimized using the high-pressure homogenization technique. Through the process of optimizing the conditions for the synthesis of solid lipid nanoparticles, it was possible to successfully select those with the properties most optimal for medical applications. The most stable solid lipid nanoparticles turned out to be those obtained based on the Softisan^®^ 601 lipid and Poloxamer 188 surfactant, and using high-pressure homogenization in a closed loop at 300 bar. In the course of the research, it was proven that by using a closed loop in high-pressure homogenization, it is possible to obtain nanoparticles with proper physicochemical properties; this was assessed on the basis of the polydispersity index (<30%), mean particle size within a 200–450 nm range, and a zeta potential of about |±30 mV|. All these observations were confirmed by DSC and XRD analyses, which showed the presence of curcumin inside the lipid nanosheets. SEM analysis showed a stable structure of solid lipid nanoparticles after curcumin application. Using confocal microscopy, it was possible to localize the active ingredient in the carrier. An analysis of the release process of curcumin allowed us to confirm its application properties. The use of spectrophotometric methods in in vitro release studies is very important for the development of new effective semi-solid drug formulations. The obtained results indicate that SLNs incorporated with curcumin can be successfully added to cosmetic formulations.

## Figures and Tables

**Table 1:** Selection of lipids versus active ingredient (curcumin) based on the experiments run at 15 min, 30 min, 1 h, 24 h, and 72 h.

Lipid + Curcumin (1:100)	Miscibility of Curcumin with Lipid *				
	15 min	30 min	1 h	24 h	72 h
Compritol^®^ 888 ATO	−	−	−	−	−
Imwitor^®^ 900 K	√	√	√	√	√
Precirol^®^ ATO 5	−	−	−	−	−
Softisan^®^ 601	√	√	√	√	√

* √ miscible; − immiscible.

**Table 2:** Summary of results for solid lipid nanoparticles incorporated with curcumin obtained, based on Softisan^®^ 601 lipid and Poloxamer 188 surfactant at 300 bar. The process was carried out in both an open- and closed-loop system in three cycles.

Sample Name	Pressure [bar]	Measurement Date	Type of Loop System	Z-Ave [nm] ± SD	PDI [%] ± SD	ZP [mV] ± SD
**SLN19**	**300**	after synthesis	open	385.30 ± 26.70	25.70 ± 3.50	|±25.10| ± 0.10
		after synthesis	closed	368.90 ± 21.00	21.70 ± 4.30	|±33.20| ± 0.10
		after 24 h	open	307.30 ± 10.30	27.70 ± 3.70	|±27.50| ± 0.30
		after 24 h	closed	386.70 ± 21.60	24.70 ± 5.40	|±30.60| ± 0.10
		after 5 days	open	206.90 ± 26.70	26.10 ± 1.00	|±23.20| ± 0.20
		after 5 days	closed	330.00 ± 10.60	25.20 ± 0.70	|±29.30| ± 0.20
**SLN20**	**400**	after synthesis	open	344.40 ± 19.20	25.10 ± 2.70	|±22.50| ± 0.30
		after synthesis	closed	407.50 ± 40.50	29.10 ± 2.90	|±27.20| ± 0.10
		after 24 h	open	208.90 ± 6.80	25.40 ± 0.90	|±22.40| ± 0.10
		after 24 h	closed	389.60 ± 43.70	29.60 ± 2.60	|±26.70| ± 0.10
		after 5 days	open	128.80 ± 10.10	23.80 ± 1.10	|±22.10| ± 0.40
		after 5 days	closed	195.40 ± 26.40	25.70 ± 1.30	|±26.30| ± 0.20
**SLN21**	**500**	after synthesis	open	389.10 ± 15.00	29.70 ± 1.40	|±23.70| ± 0.20
		after synthesis	closed	515.30 ± 37.70	26.60 ± 2.70	|±25.10| ± 0.10
		after 24 h	open	220.40 ± 6.70	26.10 ± 1.40	|±20.40| ± 0.30
		after 24 h	closed	450.50 ± 24.00	27.90 ± 2.40	|±24.80| ± 0.20
		after 5 days	open	200.20 ± 14.40	25.20 ± 3.90	|±22.90| ± 0.30
		after 5 days	closed	390.90 ± 35.40	25.40 ± 2.10	|±24.60| ± 0.20

**Table 3:** SEM images at 20, 10 and 2 μm magnification of curcumin, SLN1 and SLN19.

20 μm	10 μm	2 μm
Curcumin		
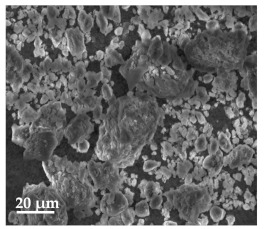	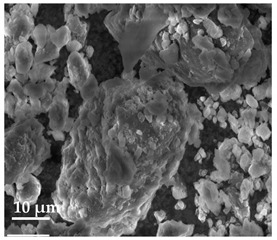	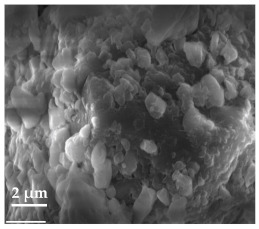
**SLN1**		
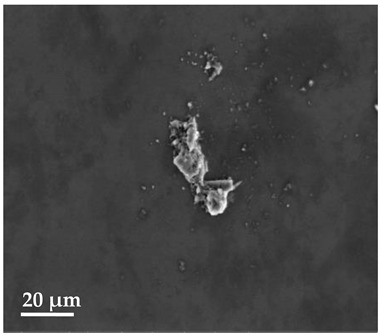	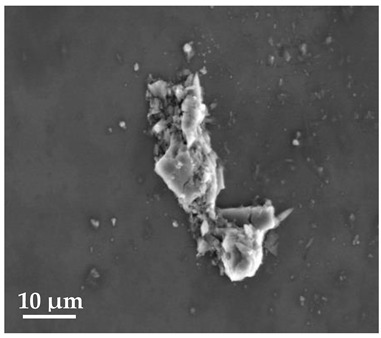	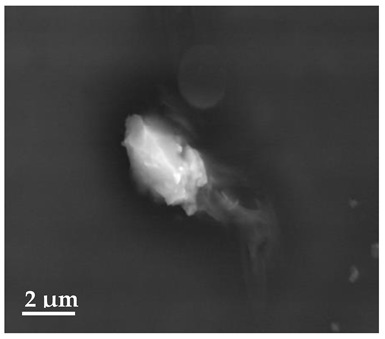
**SLN19**		
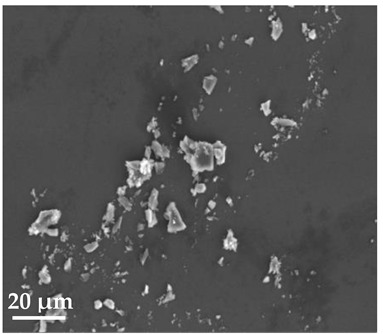	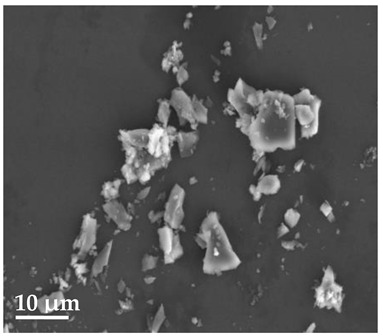	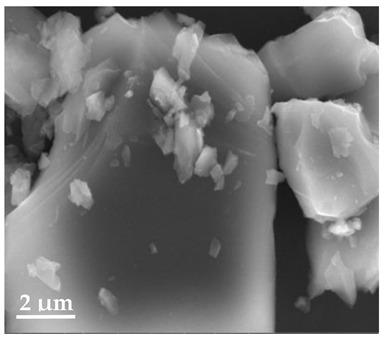

**Table 4:** Confocal microscope, i.e., (A) fluorescence, (B) bright-field, and (C) superimposed images of curcumin (left side, top), SLN1 (right side, top), and SLN19 (bottom) materials.

Curcumin 20 μm	SLN1 20 μm
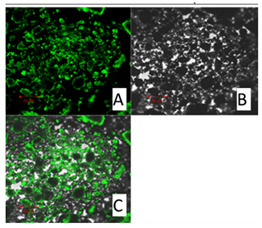	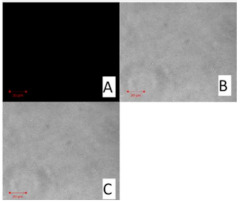
**SLN19 20 μm**	
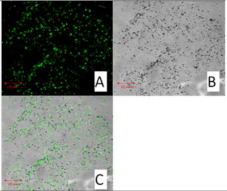	

**Table 5:** Encapsulation efficiency (EE) and loading capacity (LC) of curcumin into SLN-type lipid nanoparticles.

Sample	Incorporated Active Substance	EE ^1^ (% ± SD ^2^)	LC ^1^ (% ± SD ^2^)
**SLN19**	Curcumin	84.52 ± 0.62	12.89 ± 0.25

^1^ calculated from the equations presented in subsection “Encapsulation efficiency and loading capacity”. ^2^ SD—standard deviation.

**Table 6:** Optimization of basic formulation parameters such as lipid and surfactant type and pressure; ultrasound; and the effect of the number of homogenization cycles for obtaining solid lipid nanoparticles.

Name	Lipid	Surfactant	Technique for Obtaining	Pressure [Bar]	Number of Cycles
**Type of lipid**					
**SLN1** **SLN2**	Softisan^®^ 601	Poloxamer 188	^1^ HPH + ^2^ UT	300	3 cycles
	Imwitor ^®^ 900 K				
**Type of surfactant**					
**SLN1** **SLN3** **SLN4** **SLN5** **SLN6** **SLN7**	Softisan^®^ 601	Poloxamer 188	^1^ HPH + ^2^ UT	300	3 cycles
		Tween 21			
		Tween 40			
		Tween 60			
		Tween 80			
		Tween 81			
**Pressure**					
**SLN1** **SLN8** **SLN9**	Softisan^®^ 601	Poloxamer 188	^1^ HPH + ^2^ UT	300	3 cycles
				400	
				500	
**Effect of ultrasound treatment**					
**SLN10** **SLN11** **SLN12**	Softisan^®^ 601	Poloxamer 188	^1^ HPH + sonification 1 min	300	3 cycles
			^1^ HPH + sonification 5 min		
			^1^ HPH + sonification 10 min		
**Effect of Ultrasound and Ultra-Turrax**					
**SLN13** **SLN14** **SLN15**	Softisan^®^ 601	Poloxamer 188	^1^ HPH + sonification 10 min + ^2^ UT	300	3 cycles
				400	
				500	
**Effect of number of homogenization cycles**					
**SLN1**	Softisan^®^ 601	Poloxamer 188	^1^ HPH + ^2^ UT	300	3 cycles
**SLN16**					5 cycles
**SLN17**					6 cycles
**SLN18**					7 cycles

^1^ HPH—high-pressure homogenization; ^2^ UT—Ultra Turrax; 1 cycle—20 s.

**Table 7:** Composition of solid nanoparticles incorporated with curcumin selected by optimizing the synthesis conditions.

	Formulation Component	Mass [g]	% wt.
**Lipid phase**			
**Active ingredient**	Curcumin	0.50	1.00
**Lipid**	Softisan^®^ 601	1.00	2.00
**Water phase**			
**Surfactant**	Poloxamer 188	0.62	1.25
**Water**	Milli-Q^®^ Plus	47.87	95.75

## Data Availability

The data presented in this study are available upon request from the authors.

## Supplementary Materials

The following are available online at https://www.mdpi.com/article/10.3390/molecules27072202/s1, Table S1: Comparison of the synthesis parameters of SLNs containing different lipids, Table S2: Comparison of parameters for the synthesis of SLNs containing different surfactants, Table S3: Comparison of SLN synthesis parameters at different high pressure homogenization pressures, Table S4: Effect of sonification on emulsions synthesized at 300 bar, Table S5: Effect of ultrasound and Ultra-Turrax on emulsions, Table S6: Effect of time on the obtained solid lipid nanoparticles, Table S7: Lipids and their melting points, Table S8: Selected surfactants for the synthesis of solid lipid nanoparticles.

## Sample Availability

Samples of the compounds are not available from the authors upon request.
