# Supplementary material for: Optimization of the Conditions of Solid Lipid Nanoparticles (SLN) Synthesis
Source: Molecules. 2022 Mar 28;27(7):2202. doi: 10.3390/molecules27072202 (PMC9000502; doi:10.3390/molecules27072202)
Supplement: Supplementary file 1 [file molecules-27-02202-s001.zip › molecules-1581552-supplementary.pdf]

molecules

MDPI

Supplementary Materials

Optimization of the Conditions of Solid Lipid Nanoparticles (SLN) Synthesis

Ewelina Musielak[1], Agnieszka Feliczak-Guzik[2] and Izabela Nowak[3*]

[1] Faculty of Chemistry, Adam Mickiewicz University, 8: Uniwersytetu Poznańskiego , 61-614 Poznań, Poland; ewelina.musielak@amu.edu.pl (E.M.); agaguzik@amu.edu.pl (A.F.-G.)
* Correspondence: nowakiza@amu.edu.pl

Table S1. Comparison of the synthesis parameters of SLNs containing different lipids.

Sample Name	Measurement Date	Type of Circulation	Z-Ave [nm] ± SD	PDI [%] ± SD	ZP [mV] ± SD
**SLN1**	after synthesis	open	383.6 ± 2.6	26.3 ± 3.1	|±21.4| ± 0.1
	after synthesis	closed	255.5 ± 7.8	28.8 ± 10.9	|±29.7| ± 0.1
	after 24 h	open	248.8 ± 73.9	28.7 ± 3.8	|±20.9| ± 1.2
	after 24 h	closed	258.4 ± 10.0	29.8 ± 4.3	|±29.1| ± 0.1
	after 5 days	open	110.7 ± 40.1	27.5 ± 3.1	|±25.0| ± 0.2
	after 5 days	closed	259.5 ± 12.8	26.7 ± 1.7	|±30.1| ± 0.1
**SLN2**	after synthesis	open	388.4 ± 27.5	25.7 ± 3.9	|±26.9| ± 1.3
	after synthesis	closed	187.8 ± 17.5	33.9 ± 3.1	|±28.6| ± 0.4
	after 24 h	open	441.8 ± 31.7	26.7 ± 1.6	|±24.2| ± 0.2
	after 24 h	closed	714.9 ± 26.9	36.1 ± 1.7	|±24.1| ± 0.2
	after 5 days	open	700.4 ± 13.4	42.1 ± 3.4	|±20.4| ± 0.3
	after 5 days	closed	579.5 ± 15.8	33.4 ± 8.2	|±22.3| ± 0.4

SLN1 - Softisan®601; **SLN2** - Imwitor ®900K

Table S2. Comparison of parameters for the synthesis of SLNs containing different surfactants.

Sample Name	Measurement Date	Type of Circulation	Z-Ave [nm] ± SD	PDI [%] ± SD	ZP [mV] ± SD
**SLN1**	after synthesis	closed	255.5 ± 7.8	28.8 ± 10.9	|±29.7| ± 0.1
	after 24 h	closed	258.4 ±10.0	29.8 ± 4.3	|±29.1| ± 0.1
	after 5 days	closed	259.5 ± 12.8	26.7 ± 1.7	|±30.1| ± 0.1
**SLN3**	after synthesis	closed	151.9 ± 34.9	25.8 ± 2.2	|±29.3| ± 0.6
	after 24 h	closed	211.3 ± 6.9	23.3 ± 1.6	|±32.4| ± 0.4
	after 5 days	closed	146.3 ± 5.8	28.0 ± 3.5	|±28.5| ± 0.2
**SLN4**	after synthesis	closed	212.5 ± 12.1	25.9 ± 1.3	|±30.6| ± 0.3
	after 24 h	closed	165.7 ± 4.9	23.8 ± 2.0	|±30.2| ± 0.2
	after 5 days	closed	163.9 ± 8.5	24.2 ± 1.1	|±29.1| ± 0.1
**SLN5**	after synthesis	closed	207.7 ± 5.6	24.6 ± 0.6	|±27.7| ± 0.1
	after 24 h	closed	218.2 ± 4.9	25.5 ± 1.5	|±27.5| ± 0.3
	after 5 days	closed	209.3 ± 7.1	25.7 ± 1.5	|±25.8| ± 0.2
**SLN6**	after synthesis	closed	217.6 ± 3.7	23.4 ± 1.5	|±34.8| ± 0.2
	after 24 h	closed	204.5 ± 8.5	24.6 ± 0.9	|±33.1| ± 0.2
	after 5 days	closed	145.5 ± 9.3	26.3 ± 1.0	|±30.4| ± 0.4
**SLN7**	after synthesis	closed	180.7 ± 23.4	27.6 ± 1.3	|±26.8| ± 0.2
	after 24 h	closed	175.8 ± 28.6	26.8 ± 1.6	|±26.1| ± 0.2
	after 5 days	closed	154.2 ± 18.1	26.9 ± 1.7	|±24.6| ± 0.2

Table S3. Comparison of SLN synthesis parameters at different high pressure homogenization pressures.

Sample Name	Measurement Date	Type of Circulation	Z-Ave [nm] ± SD	PDI [%] ± SD	ZP [mV] ± SD
**SLN1**	after synthesis	open	383.6 ± 2.6	26.3 ± 3.1	ǀ±21.4ǀ ± 0.1
	after synthesis	closed	255.5 ± 7.8	28.8 ± 10.9	ǀ±29.7ǀ ± 0.1
	after 24 h	open	248.8 ± 73.9	28.7 ± 3.8	ǀ±20.9ǀ ± 1.2
	after 24 h	closed	258.4 ± 10.0	29.8 ± 4.3	ǀ±29.1ǀ ± 0.1
	after 5 days	open	110.7 ± 40.1	27.5 ± 3.1	ǀ±25.0ǀ ± 0.2
	after 5 days	closed	259.5 ± 12.8	26.7 ± 1.7	ǀ±30.1ǀ ± 0.1
**SLN8**	after synthesis	open	189.9 ± 29.2	29.1 ± 2.8	ǀ±22.5ǀ ± 0.6
	after synthesis	closed	345.2 ± 12.9	35.5 ± 9.9	ǀ±24.7ǀ ± 1.0
	after 24 h	open	228.2 ± 70.4	30.9% ± 6.6	ǀ±26.1ǀ ± 0.4
	after 24 h	closed	257.7 ± 1.3	32.6 ± 2.5	ǀ±24.3ǀ ± 0.8
	after 5 days	open	99.8 ± 17.9	23.5 ± 1.1	ǀ±22.9ǀ ± 0.8
	after 5 days	closed	590.9 ± 92.9	27.7 ± 5.3	ǀ±23.9ǀ ± 0.3
**SLN9**	after synthesis	open	102.8 ± 4.3	26.4 ± 0.4	ǀ±19.7ǀ ± 0.8
	after synthesis	closed	181.3 ± 31.1	54.7 ± 1.5	ǀ±23.8ǀ ± 0.3
	after 24 h	open	98.6 ± 14.5	27.6 ± 4.2	ǀ±19.5ǀ ± 0.7
	after 24 h	closed	238.2 ± 14.8	25.4 ± 7.6	ǀ±23.1ǀ ± 0.6
	after 5 days	open	355.5 ± 78.7	23.6 ± 2.3	ǀ±18.2ǀ ± 0.4
	after 5 days	closed	293.9 ± 18.2	37.6 ± 5.8	ǀ±21.6ǀ ± 0.2

Table S4. Effect of sonification on emulsions synthesized at 300 bar.

Sample Name	Measurement Date	Type of Circulation	Z-Ave [nm] ± SD	PDI [%] ± SD	ZP [mV] ± SD
**SLN10**	after synthesis	open	107.7 ± 3.3	28.2 ± 4.3	ǀ±19.8ǀ ± 0.2
	after synthesis	closed	315.1 ± 9.7	29.2 ± 0.8	ǀ±23.6ǀ ± 0.2
	after 24 h	open	110,4 ± 11.9	28.8 ± 3.3	ǀ±19.0ǀ ± 0.1
	after 24 h	closed	288.2 ± 22.5	31.6 ± 4.4	ǀ±23.1ǀ ± 0.4
	after 5 days	open	118,6 ± 10.4	27.4 ± 8.1	ǀ±21.2ǀ ± 0.2
	after 5 days	closed	304.0 ± 42.8	33.3 ± 8.2	ǀ±24.5ǀ ± 0.2
**SLN11**	after synthesis	open	68.8 ± 7.3	27.4 ± 2,9	ǀ±20.0ǀ ± 0.1
	after synthesis	closed	295.9 ± 14.5	39.7 ± 3.7	ǀ±23.1ǀ ± 0.2
	after 24 h	open	77.5 ± 25.1	32.4 ± 8.6	ǀ±17.1ǀ ± 0.2
	after 24 h	closed	173.7 ± 6.6	51.6 ± 1.0	ǀ±22.6ǀ ± 0.2
	after 5 days	open	112.5 ± 8.3	27.8 ± 7.2	ǀ±18.7ǀ ± 0.2
	after 5 days	closed	302.7 ± 6.0	38.7 ± 3.9	ǀ±22.7ǀ ± 1.0
**SL12**	after synthesis	open	94.0 ± 6.2	29.6 ± 4.5	ǀ±14.9ǀ ± 0.5
	after synthesis	closed	342.5 ± 22.1	24.6 ± 1.6	ǀ±20.6ǀ ± 0.2
	after 24 h	open	69.2 ± 23.4	29.4 ± 4.2	ǀ±14.7ǀ ± 1.0
	after 24 h	closed	272.6 ± 28.6	25.2 ± 4.4	ǀ±22.6ǀ ± 0.2
	after 5 days	open	624.7 ± 8.4	29.8 ± 8.9	ǀ±21.4ǀ ± 0.5
	after 5 days	closed	297.7 ± 25.3	30.2 ± 4.7	ǀ±22.8ǀ ± 0.2

Table S5. effect of ultrasound and Ultra-Turrax on emulsions.

Sample Name	Measurement Date	Type of Circulation	Z-Ave [nm] ± SD	PDI [%] ± SD	ZP [mV] ± SD
**SLN13**	after synthesis	open	91.1 ± 14.4	28.5 ± 6.7	|±16.7| ± 0.1
	after synthesis	closed	115.4 ± 10.3	48.5 ± 2.5	|±22.8| ± 1.0
	after 24 h	open	92.8 ± 16.0	29.8 ± 1.2	|±17.4| ± 0.3
	after 24 h	closed	171.6 ± 16.0	46.8 ± 2.3	|±22.3| ± 0.1
	after 5 days	open	95.2 ± 21.2	28.8 ± 4.5	|±20.0| ± 0.1
	after 5 days	closed	193.1 ± 8.1	33.9 ± 5.5	|±20.7| ± 1.0
**SLN14**	after synthesis	open	33.1 ± 4.5	18.1 ± 3.5	|±17.2| ± 0.3
	after synthesis	closed	131.7 ± 11.1	57.4 ± 6.8	|±19.4| ± 0.4
	after 24 h	open	89.0 ± 9.7	31.1 ± 6.8	|±15.5| ± 0.7
	after 24 h	closed	245.0 ± 23.3	28.8 ± 2.2	|±21.2| ± 0.2
	after 5 days	open	112.9 ± 28.9	30.6 ± 3.5	|±24.7| ± 1.0
	after 5 days	closed	212.9 ± 9.5	25.4 ± 2.9	|±20.3| ± 0.3
**SL15**	after synthesis	open	144.7 ± 18.5	27.8 ± 1.3	|±20.1| ± 0.1
	after synthesis	closed	134.3 ± 8.0	56.9 ± 7.0	|±23.8| ± 0.4
	after 24 h	open	93.3 ± 7.7	25.0 ± 3.2	|±22.0| ± 0.1
	after 24 h	closed	299.0 ± 27.1	21.7 ± 6.5	|±21.9| ± 1.0
	after 5 days	open	95.6 ± 22.4	29.8 ± 7.2	|±23.7| ± 1.0
	after 5 days	closed	209.8 ± 10.9	28.1 ± 6.5	|±21.2| ± 0.5

Table S6. Effect of time on the obtained solid lipid nanoparticles.

Sample Name	Measurement Date	Type of Circulation	Z-Ave [nm] ± SD	PDI [%] ± SD	ZP [mV] ± SD
SLN1	after synthesis	open	383.6 ± 2.6	26.3 ± 3.1	|±21.4| ± 0.1
	after synthesis	closed	255.5 ±7.8	28.8 ± 10.9	|±29.7| ± 0.1
	after 5 days	open	110.7 ± 40.1	27.5 ± 3.1	|±25.0| ± 0.2
	after 5 days	closed	259.5 ± 12.8	26.7 ± 1.7	|±30.1| ± 0.06
SLN16	after synthesis	open	128.9 ± 3.9	25.3 ± 4.6	|±30.2| ± 0.11
	after synthesis	closed	287.5 ± 11.6	27.0 ± 2.6	|±29.8| ± 0.06
	after 5 days	open	189.4 ± 5.6	27.7 ± 4.5	|±29.5| ± 0.5
	after 5 days	closed	315.8 ± 29.7	28.8 ± 1.2	|±29.3| ± 0.6
SL17	after synthesis	open	115.4 ± 4.9	24.2 ± 1.4	|±19.6| ± 0.25
	after synthesis	closed	293.6 ± 26.1	30.7 ± 6.8	|±24.6| ± 0.5
	after 5 days	open	224.7 ± 33.3	35.8 ± 3.1	|±18.0| ± 0.2
	after 5 days	closed	299.3 ± 9.9	37.8 ± 6.7	|±23.1| ± 0.2
SL18	after synthesis	open	115.8 ± 4.4	26.6 ± 1.2	|±28.6| ± 0.25
	after synthesis	closed	179.7 ± 31.8	45.8 ± 3.7	|±29.1| ± 0.06
	after 5 days	open	94.1 ± 10.9	23.7 ± 2.6	|±27.0| ± 0.1
	after 5 days	closed	292.9 ± 15.1	39.6 ± 9.4	|±28.5| ± 0.5

Table S7. Lipids and their melting points.

Trade name	Chemical name	Melting point [°C]	Producent	Country of origin
**Compritol®888 ATO**	Glycerol behemate (mixed mono-, di- and tri-)	70	Gattefossé	Poland
**Imwitor ®900K**	Glycerol monostearate, Type II	54-64	Cremer Oleo GmbH&Co. KG company	Poland
**Precirol®ATO 5**	Glycerol distearate, Type I EP	55-66	Gattefossé	Poland
**Softisan®601**	Glycerol stearate	40-45	Cremer Oleo GmbH&Co. KG company	Poland

Table S8. Selected surfactants for the synthesis of solid lipid nanoparticles.

	Surfactant	Trade name	Type of compound/HLB	Structure
	Poloxamer 188	Pluronic F68	Block copolymers/**29.0**	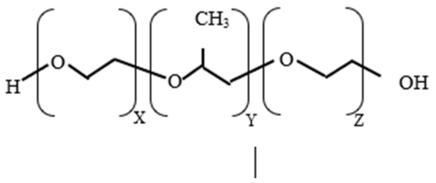
**Non-ionic surfactant**	Tween 21	Polysorbate 21	Alkyl esters/**13.3**	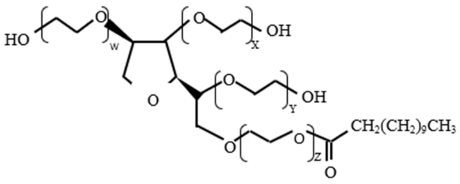
	Tween 40	Polysorbate 40	Alkyl esters/**15.6**	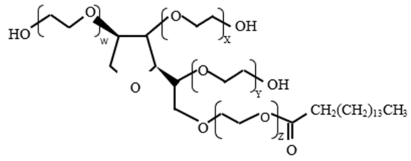
	Tween 60	Polysorbate 60	Alkyl esters/**14.9**	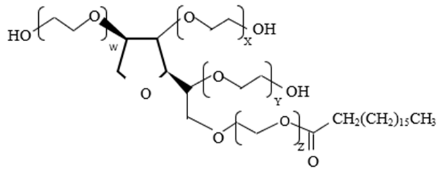
	Tween 80	Polysorbate 80	Alkyl esters/**15.0**	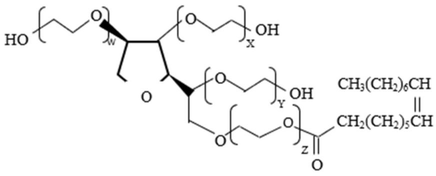

Tween 81	Polysorbate 81	Alkyl esters/**10.0**	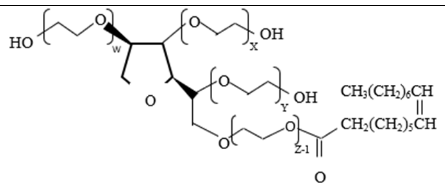
